# Clinical and Surgical Insights into Zuska Disease: A Retrospective Analysis

**DOI:** 10.3390/jpm15050170

**Published:** 2025-04-25

**Authors:** Letizia Cuniolo, Raquel Diaz, Francesca Pitto, Federica Murelli, Chiara Cornacchia, Francesca Depaoli, Marco Gipponi, Cecilia Margarino, Chiara Boccardo, Simonetta Franchelli, Marianna Pesce, Franco De Cian, Piero Fregatti

**Affiliations:** 1 Department of Surgical Sciences and Integrated Diagnostic (DISC), University of Genoa, 16126 Genoa, Italy; 2 IRCCS Ospedale Policlinico San Martino, Anatomic Pathology Unit, 16126 Genoa, Italy; 3 IRCCS Ospedale Policlinico San Martino, Breast Surgery Unit, 16126 Genoa, Italy

**Keywords:** Zuska disease, abscess, fistula

## Abstract

**Background/Objectives:** Zuska disease is a rare inflammatory condition of the mammary gland, characterized by recurrent non-puerperal abscesses in the periareolar region, often complicated by fistula formation. It predominantly affects women who smoke, particularly perimenopausal women. This study aims to investigate the clinical features, treatment outcomes, and recurrence rates of Zuska disease in a cohort of patients. **Methods:** We conducted a retrospective analysis of 19 patients diagnosed with Zuska disease and treated at the Breast Surgery Clinic of San Martino Policlinic Hospital between January 2021 and June 2024. Data were collected on demographics, clinical presentation, imaging findings, surgical interventions, intraoperative cultures, and postoperative outcomes. The types of surgeries performed, antibiotic therapy regimens, and histological findings were recorded. **Results:** The mean age of the patients was 43.8 years. The most common presentation was a painful breast mass without fistula formation (12 patients), signs of a past abscess with negative preoperative ultrasound findings (five patients), and abscesses with fistulas (two patients). Intraoperative cultures revealed a range of bacterial species. Recurrence occurred in five patients (26%), and reoperation was required in three cases. All patients were free of symptoms at follow-up, with an average recurrence time of 13.6 months. **Conclusions:** Zuska disease presents significant treatment challenges due to its recurrent nature. While surgical interventions, such as abscess drainage and ductal excision, are effective, recurrence remains common, particularly in patients with risk factors like smoking. A personalized therapeutic approach, tailored to each patient’s clinical profile, is essential to improving long-term outcomes. Early diagnosis, timely surgical management, and lifestyle modifications play a crucial role in reducing recurrence. Future studies should focus on optimizing treatment protocols and developing individualized strategies for managing comorbidities.

## 1. Introduction

Zuska disease is a rare inflammatory condition of the mammary gland, accounting for 1–2% of breast pathologies, and is characterized by recurrent non-puerperal abscesses and periareolar fistulas, primarily affecting perimenopausal women who smoke [1,2,3]. The pathophysiology involves squamous metaplasia of the lactiferous ducts, leading to obstruction, keratin accumulation, and chronic inflammation [4,5,6,7].

Clinically, it presents with localized pain, erythema, purulent discharge, and recurrent fistulas, significantly impacting quality of life due to frequent relapses and the need for repeated treatments [8,9,10]. Diagnosis relies on imaging, such as ultrasound, which reveals ductal abnormalities, but histopathology is often required to confirm the condition [2,11,12].

Management remains challenging; while antibiotics and drainage provide temporary relief, definitive treatment often requires surgical excision of the affected ducts and fistulas [12]. Smoking, hormonal factors, and comorbidities complicate healing and recurrence rates. Given these considerations, a personalized approach is indispensable, integrating microbiological, imaging, and systemic factors to achieve optimal outcomes.

This study evaluates clinical and surgical outcomes in Zuska disease, focusing on personalized management strategies to improve long-term prognosis.

## 2. Materials and Methods

We collected data from patients with Zuska disease treated at the Breast Surgery Clinic of San Martino Policlinic Hospital between January 2021 and June 2024. Data collection procedures were standardized by employing a dedicated electronic database to ensure consistency and accuracy.

Our study included a detailed analysis of both clinical and surgical variables. Preoperative clinical assessments involved recording detailed patient histories, including smoking habits, comorbidities, and any prior breast surgeries. Additionally, patients were evaluated for systemic conditions such as diabetes, autoimmune disorders, and infections like HCV and HIV, which are known to influence chronic inflammation and wound healing. Furthermore, information on body mass index (BMI) and metabolic parameters was systematically collected to assess the overall health status of each patient.

Radiological evaluations were performed using high-resolution breast ultrasound. For patients with suspected abscesses or fistulas, specific attention was given to identifying hypoechoic masses, complex cystic structures, and any signs of ductal dilation or fistula tracts. In cases without active abscesses or fistulas but with a history of prior episodes, ultrasound findings were corroborated with clinical examinations to confirm the absence of active disease. Ultrasound examinations were carried out by experienced radiologists following standardized protocols, with inter-observer variability assessments performed periodically to ensure diagnostic reliability.

Preoperative antibiotic therapy was administered based on the clinical presentation. For abscesses, broad-spectrum antibiotics were initially chosen, and the therapy was adjusted according to intraoperative culture results. Surgical interventions were tailored to the extent of the disease. The primary surgical options included abscess drainage, fistulectomy, and ductal galactophorectomy, with or without the removal of the nipple–areola complex. All surgical specimens underwent histological examination, providing critical insights into the underlying pathophysiology, including chronic inflammation, fibrosis, and granulomatous changes.

Intraoperative cultures played a pivotal role in identifying the causative pathogens, guiding postoperative antibiotic therapy.

## 3. Results

We included 19 patients in our study. The mean age of the patients was 43.8 years. Three patients had prior surgery on the same (ipsilateral) breast—two for benign pathology and one for ductal carcinoma in situ (DCIS). Five patients had a smoking history, one patient was diabetic, one was HCV+, and one was both HCV+ and HIV+. Nine patients had experienced multiple episodes of breast abscess before diagnosis.

**Figure 1:**
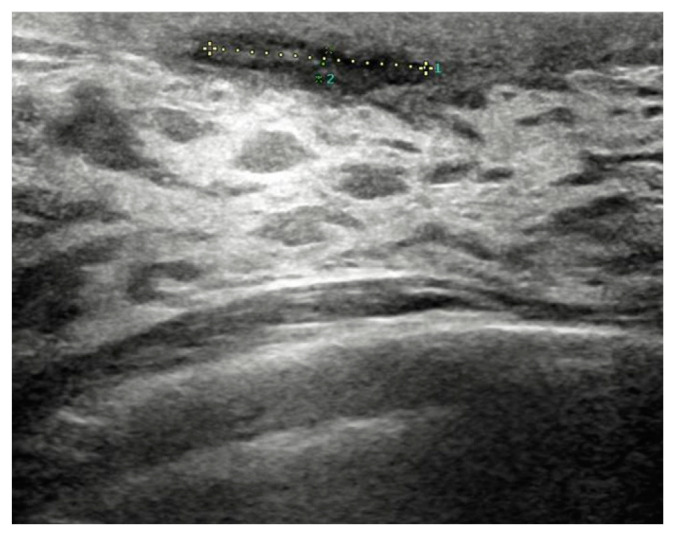
Abscess measuring approximately 16 mm × 2.5 mm in extent, without a liquid component, located in the retroareolar region.

**Figure 2:**
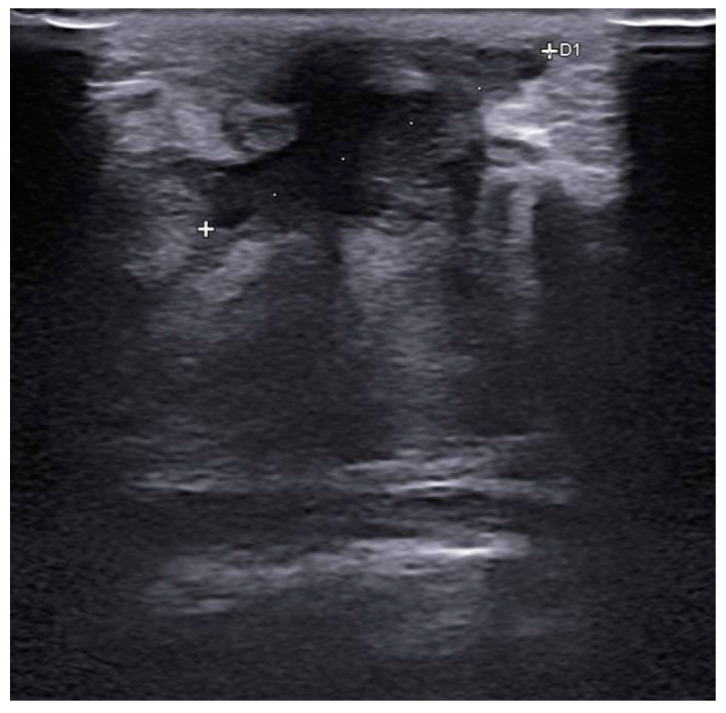
Abscess with a maximum extent of 25 mm, reaching close to the subcutaneous tissue.

**Figure 3:**
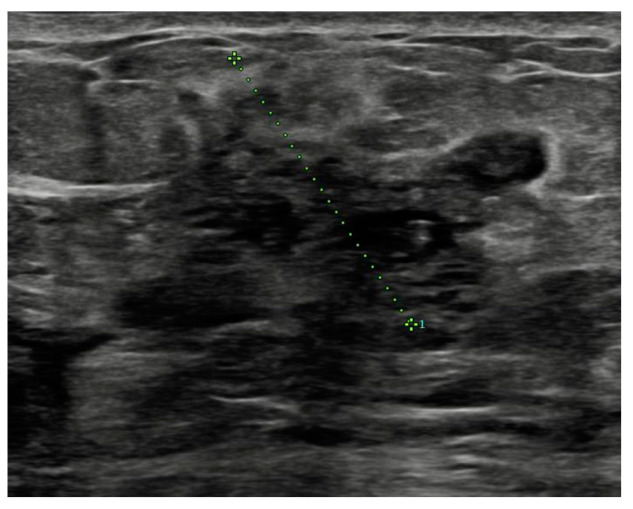
Abscess with an overall extent of 6 cm × 3.5 cm.

**Figure 4:**
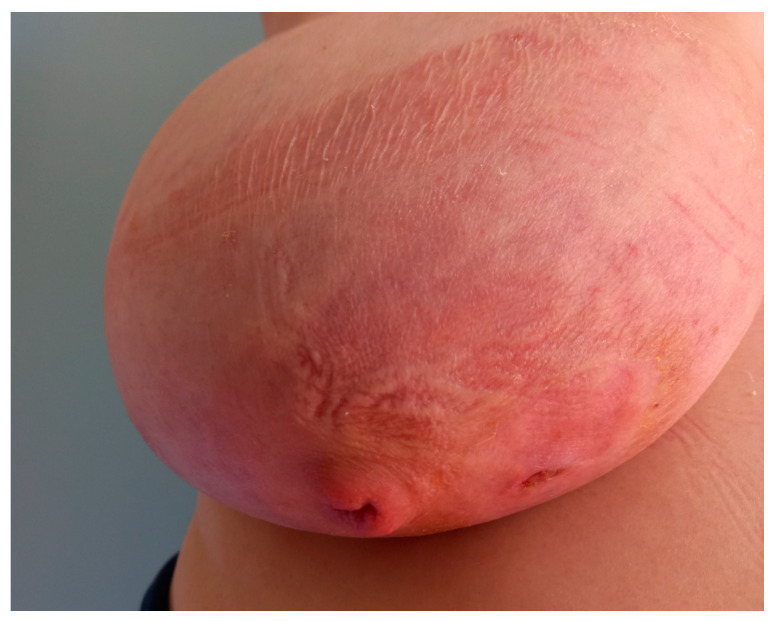
Abscess with a fistula in the periareolar region.

Before surgery, all patients underwent breast ultrasound to assess the presence of an abscess or fistula. Regarding clinical and radiological presentation, 12 patients presented with an abscess in the form of a painful and tender breast mass, with tense and reddened skin but no cutaneous fistula, confirmed by ultrasound (Figure 1, Figure 2 and Figure 3); two patients presented with an abscess associated with periareolar fistula (Figure 4), visible on breast ultrasound; five patients showed signs of a past abscess with negative preoperative ultrasound findings, and among them, one had a fistulous tract without purulent discharge (Table 1). The location of abscesses, fistulas, and their sequelae was the periareolar area in all patients. Additionally, four patients presented with bilateral symptoms. For patients with active abscesses at surgery, the mean size of the abscess cavity was 28.7 mm.

All patients underwent surgery for the treatment of Zuska disease. Patients who, at the time of surgery, presented only with sequelae of an abscess and/or fistula received antibiotic prophylaxis with 2 g of Cefazolin at anesthesia induction. This prophylactic regimen was standardized across the cohort to minimize perioperative infections. Patients with an abscess, with or without a fistula, at the time of surgical planning were treated with broad-spectrum preoperative antibiotic therapy for at least one week (Amoxicillin/Clavulanic Acid or Trimethoprim/Sulfamethoxazole).

Regarding the type of surgery, five patients underwent abscess drainage alone, two underwent fistulectomy, two underwent fistulectomy combined with abscess drainage, five underwent ductal galactophorectomy (the removal of lactiferous ducts), and four underwent ductal galactophorectomy combined with abscess drainage. In one patient, bilateral excision of the nipple–areola complex was required to resolve the clinical condition. The patients who underwent fistulectomy and ductal galactophorectomy without abscess drainage were the seven patients presenting only with sequelae of a breast abscess, with negative preoperative ultrasound findings. The surgical decision-making process was based on a comprehensive evaluation of clinical findings and imaging data, ensuring that the most appropriate intervention was selected for each patient. A multidisciplinary team discussion, including surgeons, radiologists, and infectious disease specialists, was held to optimize treatment strategies. The decision to perform abscess drainage versus ductal galactophorectomy was based on the clinical presentation and the extent of duct involvement (Table 2).

During all surgeries, a sample was taken for culture testing, which was negative in nine patients and positive for

• *Staphylococcus aureus* (multisensitive) in one patient;
• *Proteus mirabilis* in one patient;
• *Actinomyces neuii* in one patient;
• *Staphylococcus warneri*, *Staphylococcus epidermidis*, and *Enterococcus faecalis* in one patient;
• *Staphylococcus lugdunensis* and *Prevotella bivia* in one patient;
• *Staphylococcus lugdunensis* alone in one patient;
• *Mycobacterium gordonae* in one patient;
• *Mycobacterium chelonae* in one patient;
• *Finegoldia magna* and *Anaerococcus vaginalis* in one patient;
• *Prevotella* spp. and *Finegoldia magna* in one patient.

All patients with an abscess at the time of surgery were treated with broad-spectrum postoperative antibiotic therapy, adjusted based on the intraoperative culture results. The two patients who tested positive for mycobacteria (*Mycobacterium gordonae* and *Mycobacterium chelonae*) were treated with Ethambutol, Azithromycin, and Levofloxacin. These cases were managed in collaboration with infectious disease experts to ensure adherence to the recommended therapeutic protocols for atypical mycobacterial infections.

The histopathological evaluation was conducted by experienced pathologists using standardized criteria. Definitive histological examination revealed fibrosis and chronic inflammation in all patients. Additionally, five patients showed lymphoplasmacytic inflammation, five granulomatous inflammation (including one patient positive for *Mycobacterium gordonae* and one for *Mycobacterium chelonae*), three had papillary hyperplasia, two had ductal hyperplasia, two had papillary and ductal hyperplasia, and two had an intraductal papilloma (Table 3).

Recurrence was defined as the reappearance of clinical or radiological signs of disease after an initial period of resolution, and occurred in five patients (26%). Among them, two were treated conservatively with antibiotic therapy, while three required reoperation combined with antibiotic therapy.

Of the three patients who underwent reintervention:

• The first underwent quadrantectomy with abscess drainage, fistulectomy, and bilateral mammoplasty.
• The second underwent nipple-sparing mastectomy with reconstruction using an expander.
• The third underwent abscess evacuation with removal of the nipple–areola complex.

The average time between the first surgery and recurrence was 13.6 months. For the three reoperated patients, the average time between the first and second surgeries was 10.6 months.

Currently, all patients are free of symptoms related to Zuska disease. Follow-up evaluations, including clinical examinations and imaging studies, confirming the long-term resolution of symptoms and the effectiveness of the therapeutic interventions.

## 4. Discussion

Zuska disease is often associated with chronic inflammation of the mammary ducts (periductal mastitis), with cigarette smoking being the main risk factor, as it promotes duct obstruction and subsequent infection [3]. Cigarette smoke is known not only to impair wound healing but also to induce chronic inflammatory changes at the cellular level, thereby creating an environment that promotes ductal obstruction and infection [3,13]. Chronic inflammation leads to a metaplastic transformation of the ductal epithelium, which results in keratin overproduction and accumulation within the ducts, leading to their obstruction and eventual dilation [13]. The subsequent extravasation of keratin and inflammatory debris incites a robust inflammatory response that may culminate in abscess formation and, ultimately, the development of fistulae [8,9,14]. This cascade establishes a vicious cycle wherein persistent inflammation further promotes metaplastic changes, thereby predisposing patients to recurrent infections even after initial treatment appears successful. It is important to note that this cycle is often exacerbated by factors such as repeated local trauma or ongoing exposure to environmental irritants [8,9]. Although smoking is recognized as one of the main risk factors for Zuska disease, our analysis revealed that only 26% of patients in the study group (5 out of 19) had a history of smoking. This discrepancy may reflect a different distribution of risk factors in our cohort compared to those reported in previous studies, and it may suggest that Zuska disease, at least in certain subgroups, can develop even in the absence of the primary risk factors. Therefore, adopting a personalized approach to the management of Zuska disease, one that considers the individual characteristics of each patient, is crucial.

Other risk factors include bacterial infections, notably those caused by atypical organisms and mycobacteria, hormonal fluctuations, local trauma, and autoimmune conditions [14,15,16]. In addition to these factors, external elements such as nipple piercing, aggressive hair removal, and minor skin disruptions in the areolar region have been implicated as potential contributors to disease pathogenesis, as potential entry points for pathogens, exacerbating local inflammation local inflammatory responses [5,12]. Comorbidities such as diabetes, HCV, or HIV infection further complicate the clinical context [17]. For instance, diabetes not only compromises immune defenses and delays wound healing but also creates a metabolic environment that favors persistent infections and recurrent abscess formation [3,14,15,18,19]. These systemic conditions, when combined with deleterious lifestyle factors like smoking, amplify the local disorder and contribute to a more aggressive clinical course. In particular, the interplay between diabetes and smoking can create a particularly challenging clinical scenario, as both conditions are independently linked to impaired immune function and increased susceptibility to infection [14,18,19].

Therefore, the treatment of Zuska disease should adhere to the principles of personalized medicine, with therapeutic approaches tailored to each patient’s unique clinical profile. Individual factors, including age and comorbidities, and lifestyle habits, such as smoking, play a critical role in determining both the clinical presentation and the response to treatment [15]. Vitamin A deficiencies have also been linked to the development of squamous metaplasia underlying Zuska disease [13]. Thus, a comprehensive management plan should focus not only on surgical and medical interventions but also incorporate targeted smoking cessation programs and nutritional support [5]. Moreover, rigorous management of diabetes through strict glycemic control is essential, as improved metabolic control can enhance wound healing and reduce recurrence risk [3,15,18,20].

Another essential aspect of personalized management in Zuska disease is intraoperative microbiological analysis. The most commonly found bacteria in nonpuerperal breast abscesses belong to the Staphylococcus and Streptococcus species. Other microorganisms associated with nonpuerperal breast abscesses include Enterobacteriaceae, Corynebacterium, Escherichia coli, and Pseudomonas among aerobes, as well as anaerobes such as Peptostreptococcus, Propionibacterium, and Bacteroides [20,21]. In rare cases, the microorganism Prevotella bivia has been isolated [22,23]. In addition to bacteria belonging to the aforementioned classes, in our study, we also identified in patients without comorbidities some microorganisms belonging to the class of mycobacteria, specifically *Mycobacterium gordonae* and *Mycobacterium chelonae*. Mycobacteria are rarely the cause of breast abscesses in healthy women, but they should be considered as potential etiological agents, even in the absence of immunosuppression or trauma [15]. Thus, our work highlights the importance of obtaining intraoperative cultures to accurately identify pathogens and tailor postoperative antibiotic therapy. Tailored antibiotic treatment not only enhances the likelihood of complete bacterial eradication but also reduces the risk of antimicrobial resistance, a growing concern in the management of soft tissue infections [14,15,16]. Evidence from previous studies consistently supports the notion that culture-directed antibiotic regimens lead to better outcomes compared to empirical approaches, particularly in cases of recurrent disease where mixed or resistant flora may be present [14,15,16,17,24]. Such detailed microbiological analysis is crucial for guiding subsequent therapy and preventing further recurrence.

Furthermore, the long-term success of surgical intervention in Zuska disease is intricately linked to the effective management of underlying risk factors. As previously emphasized, patients with diabetes or autoimmune disorders benefit substantially from specialized perioperative care, which includes not only stringent glycemic control but also, when indicated, immunomodulatory therapies that help temper the inflammatory response [14,15,16,17,18,19]. Surgical strategies aimed at complete excision of the abnormal lactiferous ducts, as well as any associated fistulous tracts, have been shown to markedly reduce the risk of recurrence [25]. Incomplete resection of the pathological ducts, especially in the presence of ongoing risk factors like persistent smoking, often increases the risk of relapse, potentially requiring further surgical interventions [23,25]. The chronicity and complexity of nonpuerperal subareolar abscesses, commonly seen in Zuska disease, often require a more aggressive surgical approach compared to lactational abscesses due to their tendency for extensive ductal involvement and complex fistula formation [2].

The management of Zuska disease is undoubtedly multifaceted and requires a multidisciplinary approach. While antibiotics remain vital for controlling acute infections, the decision between conservative management options, such as ultrasound-guided needle aspiration, and more definitive surgical intervention is often determined by the extent of ductal involvement and the presence of fistulae [25,26,27,28,29]. Advances in imaging techniques have improved our ability to delineate the full extent of disease preoperatively, facilitating precise measurement of abscess size, identification of loculated collections, and detection of subtle ductal abnormalities, thereby aiding in the formulation of an optimal treatment plan [12,25,29].

When necessary, surgical treatment may require procedures such as abscess drainage, fistulectomy, or galactophorectomy, performed either alone or in combination, depending on the severity of duct involvement and the presence or absence of a fistula [12,29]. Recent surgical series have reported that complete duct excision, which involves removing the entire affected ductal system, can significantly lower recurrence rates, thereby offering a more definitive solution for patients with chronic or recurrent disease [13]. In our study, we observed a recurrence rate of 26%, consistent with the literature. Recurrence rates range from 70–80% at 6 months when only fistulotomy is performed [30] to 10–20% when fistulectomy and abscess drainage are carried out [31]. This variation may be attributed to factors such as incomplete resection of the lactiferous ducts, persistent smoking habits, or failure to address the risk factors [21,32]. Nonpuerperal subareolar abscesses have a higher recurrence rate compared to lactational breast abscesses and often require repeated drainage or surgical interventions [22,32]. This underscores the importance of an individualized surgical approach, where the technique used should be tailored to the patient’s disease extent and overall health condition.

Furthermore, the literature suggests that early intervention, along with a thorough preoperative assessment, can modify the progression of the disease in patients with Zuska disease [8,9]. Early recognition of risk factors, followed by prompt initiation of a tailored treatment regimen, may prevent the progression to more severe forms of the disease, including extensive fistulization and ductal destruction [8,9]. Preventive strategies, such as patient education on the importance of smoking cessation and strict management of comorbid conditions, are vital components of a long-term management plan [5].

Our study has several limitations, the most significant of which relates to the sample size. With only 19 patients included, the sample is not large enough to draw generalizable conclusions or to apply complex statistical methods that require a larger number of participants to achieve adequate power. The small sample size makes it difficult to reliably identify subtle trends or significant relationships between variables. Moreover, the limited sample size may have affected the ability to detect significant differences in clinical outcomes, thus limiting the interpretability of the data. We have chosen to address this limitation through a descriptive analysis, which, while not allowing for robust statistical inferences, still provides a useful overview of the patients’ characteristics and the observed outcomes. Despite this, our research offers an important data foundation for future studies that could include a larger sample size, thus increasing statistical reliability.

In summary, the pathogenesis of Zuska disease is multifactorial, involving a complex interplay between ductal squamous metaplasia, chronic inflammation, and infection, with cigarette smoking and metabolic comorbidities serving as major contributing factors [3,14,19]. A comprehensive treatment strategy that integrates personalized medicine, through tailored antibiotic regimens based on intraoperative culture data, meticulous surgical intervention, and proactive management of systemic risk factors, is essential for improving long-term outcomes and minimizing recurrence [5]. This integrated and multidisciplinary approach enables the effective management of both the local pathological processes within the breast and the systemic conditions that predispose patients to recurrent disease. In this way, more effective and lasting solutions can be offered to patients affected by this challenging condition, ultimately improving their quality of life.

## 5. Conclusions

Zuska disease can be challenging to treat due to its recurrent nature and complex pathogenesis. In cases where conservative management, including antibiotic therapy and percutaneous drainage, is ineffective, surgical intervention offers a promising avenue for resolution. It is important to note that, in some cases, repeated drainage or multiple surgeries may be required, depending on the severity of duct involvement and the presence of fistulae. Early diagnosis and timely surgical management, supported by advanced imaging techniques, are critical for accurately delineating disease extent and planning the optimal intervention. Technological advancements in imaging have further refined our ability to assess disease progression and guide treatment decisions. Additionally, addressing modifiable risk factors, especially smoking cessation, is vital to reducing recurrence rates.

By adopting a personalized approach that considers each patient’s unique clinical profile, including comorbidities such as diabetes, nutritional status, and lifestyle habits such as smoking, physicians can significantly improve patient outcomes and reduce the risk of recurrence. The incorporation of targeted smoking cessation programs and improved metabolic control, particularly in diabetic patients, can contribute to a more favorable treatment outcome. Tailoring antibiotic regimens based on intraoperative culture data and employing accurate surgical techniques to ensure complete duct excision are key components of this strategy.

Future research should focus on developing standardized protocols that integrate the principles of personalized medicine, ensuring that therapeutic strategies are not only evidence-based but also adaptable to individual patient needs. Moreover, studies should investigate the long-term benefits of combining early intervention, comprehensive risk factor management, and targeted surgical procedures to further enhance outcomes in patients with Zuska disease. A multi-center, longitudinal study with a larger sample size would be invaluable in further validating these approaches and refining treatment protocols.

## Figures and Tables

**Table 1:** Preoperative clinical and radiological presentation.

Clinical and Radiological Presentation	Number of Patients
Mammary abscess without fistula	12
Sequelae of past abscess	5
Mammary abscess with fistula	2

**Table 2:** Surgical Approach.

Surgical Procedure	Number of Patients
Abscess drainage	5
Fistulectomy	2
Fistulectomy with abscess drainage	2
Galactophorectomy	5
Galactophorectomy with abscess drainage	4
Bilateral nipple-areola complex removal	1

**Table 3:** Histopathological findings.

Histopathological Findings	Number of Patients
Fibrosis and chronic inflammation	19
Lymphoplasmacytic inflammation	5
Granulomatous inflammation	5
Papillary hyperplasia	3
Ductal hyperplasia	2
Papillary and ductal hyperplasia	2
Intraductal papilloma	2

## Data Availability

The data presented in this study are available in this article.
